# Patients with Modic type 2 change have a severe radiographic representation in the process of lumbar degeneration: a retrospective imaging study

**DOI:** 10.1186/s13018-019-1355-y

**Published:** 2019-09-05

**Authors:** Mindong Lan, Yufu Ou, Chenglong Wang, Wei Wei, Xianwei Lu, Jianxun Wei, Xiaoping Mu

**Affiliations:** 1 Department of Orthopaedics, Wuming Hospital of Guangxi Medical University, Nanning, China; 2 Department of Orthopaedics, People’s Hospital of Guangxi Zhuang Autonomous Region, Nanning, China

**Keywords:** Endplate signal changes, Modic changes, Lumbar degeneration, Lumbar sagittal parameters

## Abstract

**Background:**

There are few studies to investigate changes in imaging parameters of Modic changes (MCs). The imaging studies examining the distinctions in the lumbar sagittal parameters between MCs and lumbar disc degeneration (LDD) are still lacking. The purpose of this study was to identify the differences in the lumbar sagittal parameters among patients for LDD with/without Modic type 2 change (MII).

**Methods:**

A total of 208 patients with lumbar degenerative disease from January 2017 to August 2018 volunteered for this study. Sixty-two patients with MII were used as the MC group. The other 146 patients served as the disc degeneration (DD) group. The DD scores and sagittal parameters were measured on magnetic resonance imaging (MRI) and X-ray by using Surgimap software.

**Results:**

The prevalence of MII for patients with degenerative lumbar diseases in this study was 29.81%, primarily located at L5/S1. There were significant differences in lumbar lordosis (LL) and sacral slope (SS) between these two groups (*P* < 0.05). Similarly, the significant decrease in intervertebral height index (IHI) was found at L3-S1 in the MC group, compared with the DD group (*P* < 0.05). However, a significant difference in intervertebral angle (IVA) was observed only at L5/S1 (*P* < 0.05). The MC group had the smaller endplate concave angle (ECA) than the DD group from L3 caudal endplate to S1 cranial endplate (*P* < 0.05).

**Conclusions:**

MII has a severe radiographic representation in the process of lumbar degeneration than patients without MII, and the overconcentration of load caused by the smaller LL, SS, and IVA may be a reasonable explanation to answer why MCs are more common at the L5/S1.

## Introduction

The endplates between vertebral bone and intervertebral disc are the important elements in the structures of the lumbar spine, which are composed of thin hyaline cartilage with an average thickness of 0.6 mm [1, 2]. The anatomical structure of the endplate determines its important biomechanical role in walking upright humans. With the development of medical imageology, vertebral endplate can be clearly observed on MRI. Subsequently, Modic et al. [3, 4] first described the classification and histological features of the vertebral endplate and its adjacent bone marrow signal changes.

Currently, Modic changes (MCs) have been considered as an important feature of spinal degeneration on MRI. Although the etiology of MCs remains unclear, abnormal gene fragment may be the basis of the occurrence of MCs [5, 6]. Moreover, the microfracture of endplate caused by degenerative spinal structure [7] and repetitive “fatigue” loading [8] is the key link of MCs. Besides, endplate and intervertebral disc damage caused by biomechanical imbalances can also provide important conditions for subsequent immune responses [9]. Obviously, the instability of the spinal structure and sagittal imbalance play the important roles in the occurrence of MCs [10].

Normal spinal and pelvic curvature can keep the body position in the best balance. This balance formed by the interaction between the spine and the pelvic directly affects the mechanical properties of the intervertebral disc and its adjacent bone and soft tissue [11]. Once the sagittal lines of gravity change, the spinopelvic balance will be broken and the human body also will need an increase in energy consumption without external support accordingly [12]. Therefore, the sagittal imbalance may be a potential contributing factor for the development of lumbar degenerative diseases. Lumbar sagittal parameters, including but not limited to lumbar lordosis and lumbosacral angle, have been proved to be linked to lumbar disc degeneration (LDD) [13, 14]. We speculate that MCs may be associated with the sagittal imbalance and LDD. Because degenerative sagittal imbalance can alter the axial stress of spinal motion unit, then, long-term axial stress can undoubtedly lead to the lesions of endplate and intervertebral disc. Meanwhile, deformation of intervertebral disc and endplate can in turn aggravate the sagittal imbalance of lumbar spine [15].

To our best knowledge, no studies that assessed and compared lumbar sagittal parameters in patients with LDD alone and those with LDD plus MCs were retrieved from the literature. Due to almost all patients with MCs accompanied by LDD that was rated as grade 3 or more in the Pfirrmann classification, we used subjects with LDD alone (grades 3–5 in the Pfirrmann classification) as a control group in order to assess whether there was any difference in the sagittal parameters between patients with Modic type 2 change (MII) and with LDD alone.

## Materials and methods

### Study design and population

**Fig. 1:**
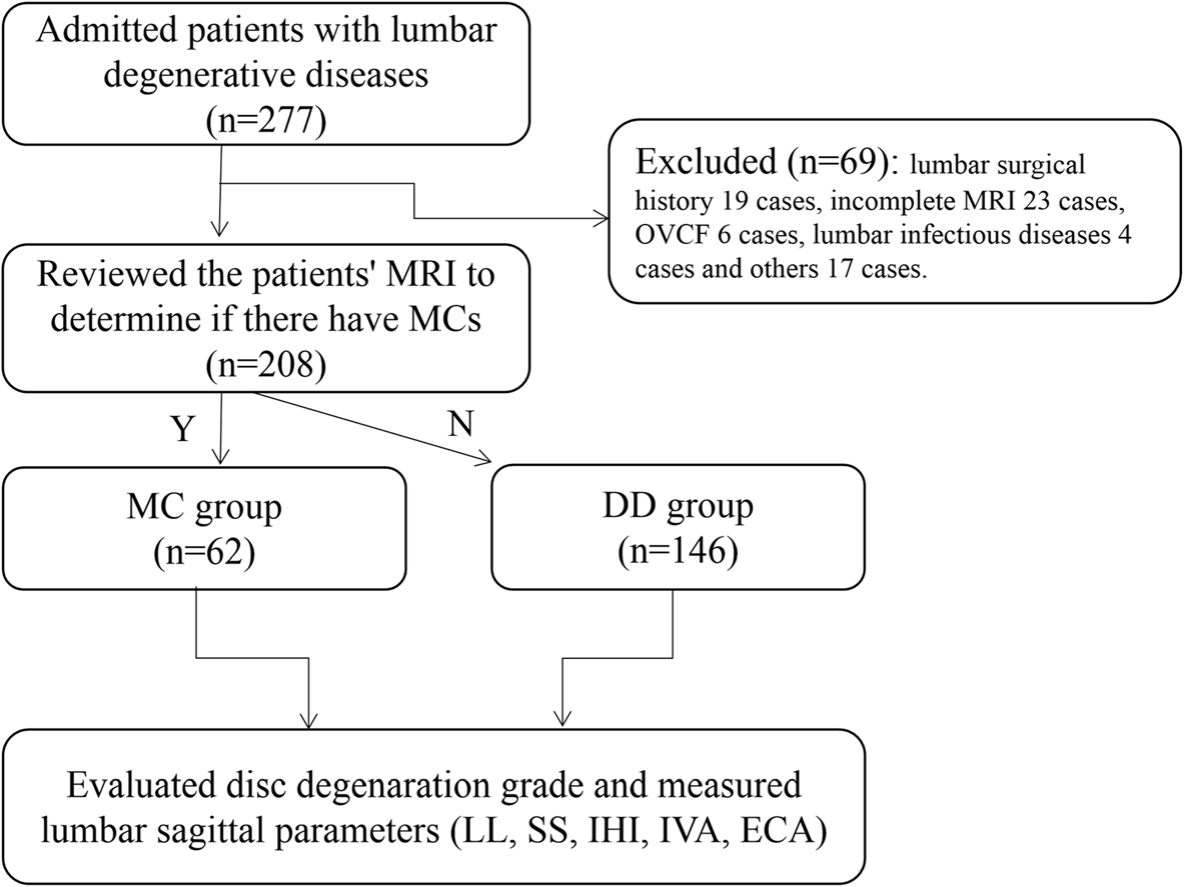
The recruitment and selection process of the study population. OVCF, osteoporotic vertebral compression fracture

In this study, we collected the data of patients who almost underwent low back pain caused by lumbar degenerative diseases from January 2017 to August 2018 in our hospital. The recruitment and selection process of the study population is shown in Fig. 1. We considered the including patients who met the following criteria in this study: (1) patients with MII or LDD and disc degeneration (DD) grade rated as one of grades 3 to 5 in the Pffirmann classification, (2) the age of patient more than 18 years old, (3) shooting posture met the standards and had the complete and clear information on lumbar X-ray (standing position) and MRI (supine position), and (4) patients who volunteered to participate in this study on the premise of knowing the study details and signed the relevant informed consent.

The exclusion criteria were as follows: (1) the previous history of lumbar surgery; (2) accompanied with spinal deformities and hip or knee diseases; (3) accompanied with the diseases that can lead to the destruction of the spinal structure, such as spinal tuberculosis, infection, tumor, and ankylosing spondylitis; (4) osteoporotic vertebral compression fracture with loss of vertebral height; (5) abnormal gait and posture caused by disability of lower extremity or other diseases; and (6) an inability to understand the study protocol after explanation.

The study was conducted in strict accordance with the principles of the Declaration of Helsinki. And this study is a part of the project entitled “the imaging study of intervertebral disc and endplate degeneration for patients with cervical and lumbar degenerative diseases” approved by authors’ Institutional Ethical Committee in 2016 (no. 2016-12). The medical information of all subjects in this study stored in the hospital’s electronic medical system can only be used with patients’ informed consent.

### X-ray and MRI examination

For all patients, the X-ray and MRI examination were performed in accordance with the standard procedures and the range of scanning was L1-S1. The detailed parameters of MRI were as follows: sagittal T1-weighted (time of repetition/echo time = 400 ms/8 ms) and T2-weighted images (time of repetition/echo time = 3000 ms/100 ms), slice thickness of sagittal scanning = 4 mm, interval = 0.4 mm, and FOV (field of view) 512 × 512 pixel.

All the images required for measurement in this study were copied from the electronic imaging system workstation of our hospital. Two doctors with more than 3 years of experience in spinal surgery evaluated independently patients’ MRI and X-ray by using Surgimap software (version 2.2.15, Nemaris Inc., USA) on the premise of single blindness.

### Classification of LDD

According to the Pfirrmann classification criteria [16], LDD was classified into 5 grades on the T2-weighted imaging. Since MCs usually were observed at the lumbar segment with more than grade 3 of LDD, this study included only those patients whose DD grades were rated as grades 3 to 5, in order to maintain comparability of the baseline between two groups.

### Evaluation of MCs

**Fig. 2:**
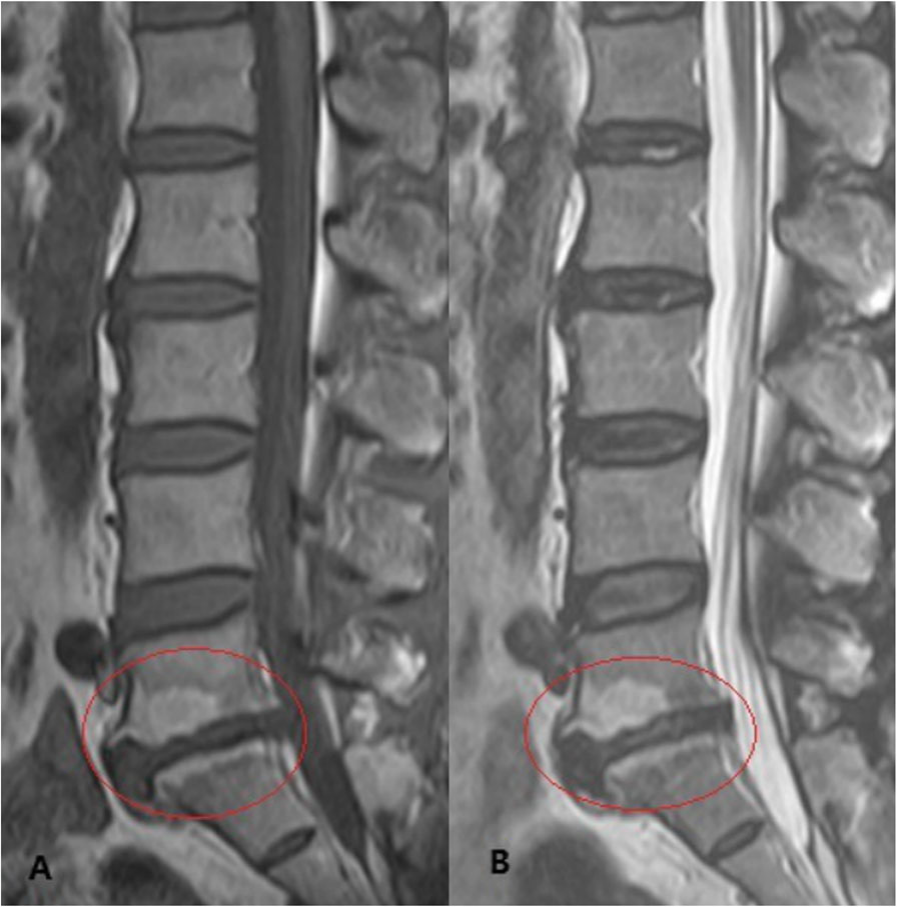
Modic type 2 change. **a** Hyperintensity on T1-weighted images at the L5/S1. **b** Hyperintensity on T2-weighted images at the L5/S1

According to the MC classification criteria [3, 4], three types of MCs have been described based on their appearance on T1-weighted and T2-weighted images. MII (Fig. 2) with the hyper-intensity on both T1-weighted images and T2-weighted images, being the most common among these three types, was considered to be associated with fatty degeneration (yellow marrow replaced red marrow).

### Measurement of sagittal parameters

**Fig. 3:**
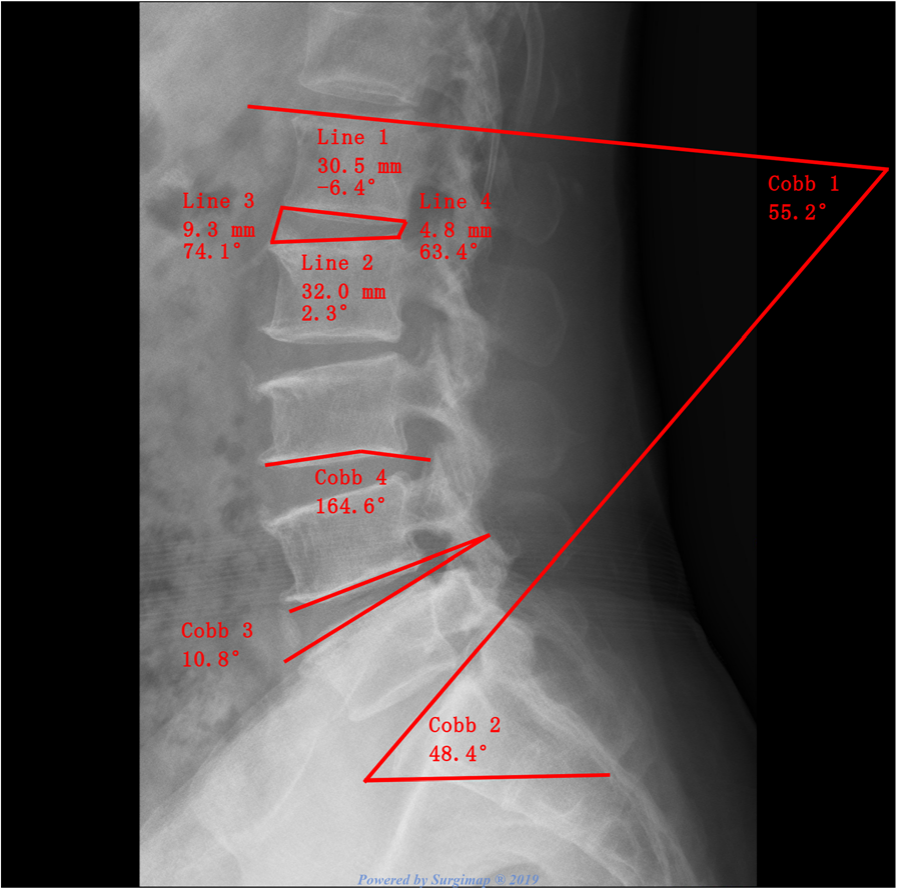
Measurements of lumbar sagittal parameters. Cobb 1, lumbar lordosis (LL); Cobb 2, sacral slope (SS); Cobb 3, intervertebral angle of L4/5 (IVA); Cobb 4, L3 caudal endplate concave angle (ECA). IHI = (line 1 + line 2)/(line 3 + line 4) × 100%

The measuring parameters in this study were as follows (Fig. 3): (1) lumbar lordosis (LL), formed by two oblique lines that were drawn through and parallel to the L1 and S1 upper endplate, respectively; (2) sacral slope (SS), assessed through the intersection of lines parallel to the sacral base and ground; (3) intervertebral height index (IHI) = (the height of anterior disc + the height of posterior disc)/(superior disc width + inferior disc width); (4) intervertebral angle (IVA), which was the intersection of two lines through and parallel to the upper and lower endplates, respectively; and (5) endplate concave angle (ECA), formed by the lines that were drawn from the bottom and summit of arc along to the endpoints.

### Statistical methods

We used the SPSS software (version 22.0) to analyze the data included in this study. Mean ± standard deviations (*x* ± *s*) were the main manifestations of data results, and percentage (%) was used to show the epidemiological characteristics of MII. The interobserver reliability was evaluated using intraclass correlation coefficient (ICC). In the MC group, chi-square test was performed for the incidence of MII between the different genders. The Mann-Whitney *U* test was carried out to analyze the LL, SS, IHI, IVA, and ECA between the two groups. Statistical significance was considered when *P* value was < 0.05.

## Results

### Patient demographics and prevalence of MC

We reviewed the MR images of 208 patients. 29.81% (62/208) of patients with MII on the basis of LDD were used as the MC group. One hundred forty-six patients served as the DD group. The average age of patients in the MC group and DD group was 51.65 ± 11.34 and 49.08 ± 13.55, respectively, without the significant difference between both groups (*P* = 0.193). No significant difference in gender was found between these two groups (*P* > 0.05). In addition, there was no significant difference in the Pfirrmann DD score between the LDD and MC groups (*P* > 0.05). However, the incidence of MII in female 64.52% (40/62) was higher than in male 35.48% (22/62). The most common distribution of MII was L5/S1 (40 cases).

### Interobserver reliability

Interobserver reliability was counted between two reviewers for the classification of DD, MCs, and their types, as well as for the measurement of the sagittal parameters. The excellent agreement was observed for the classification of DD (ICC value 0.84), MCs (ICC value 0.93), and their types (ICC value 0.89). There was a substantial agreement between the two reviewers on LL (ICC value 0.78), SS (ICC value 0.71), IVA (ICC value 0.75), and ECA (ICC value: 0.69). The two reviewers had moderate agreement on IHI (ICC value 0.58).

### Sagittal parameters

Comparison of the LL, SS, IHI, and IVA between the two groups is shown in Table 1. The LL value in the MC group and DD group was 33.30 ± 12.23 and 39.03 ± 11.30, respectively. The SS value was 30.70 ± 8.38 and 35.21 ± 7.67. There were the significant differences in LL (*P* < 0.01) and SS (*P* < 0.01) between these two groups. Similarly, the significant decreases in IHI were found at the segments of L3/4 (*P* < 0.01), L4/5 (*P* < 0.01), and L5/S1 (*P* < 0.01) in the MC group, compared with the DD group. The above parameters (LL, SS, and IHI) indicated that the MC group had more severe degeneration than the DD group. The lower the lumbar levels were, the greater the IVA had. The IVAs located at L3-S1 in the MC group were smaller than those of the DD group. However, a significant difference in IVA was observed only at L5/S1 (*P* < 0.01).

**Table 1:** Comparison of LL, SS, IHI, and IVA between both groups

Items	DD	MC	*U* value	*P* value
LL (°)	39.03 ± 11.30	33.30 ± 12.23	3.12	< 0.01
SS (°)	35.21 ± 7.67	30.70 ± 8.38	3.966	< 0.01
IHI				
L3/4	30.01 ± 3.96	25.54 ± 6.29	2.805	< 0.01
L4/5	31.92 ± 5.22	28.03 ± 5.79	2.902	< 0.01
L5/S1	33.66 ± 5.39	29.73 ± 7.19	3.353	< 0.01
IVA (°)				
L3/4	6.10 ± 2.63	5.02 ± 2.93	0.984	> 0.05
L4/5	6.49 ± 3.80	6.54 ± 3.97	− 0.095	> 0.05
L5/S1	12.37 ± 4.40	9.52 ± 4.94	3.081	< 0.01

Comparison of ECA between the MC group and DD group is shown in Table 2. Quantitative analysis of ECA revealed that compared with the DD group, the significant decreases in ECA were found at the L3-S1 caudal and cranial endplate (*P* < 0.05) in the MC group, indicating that MII was a contributing factor to endplate lesion.

**Table 2:** Difference of ECA between the two groups

Items	DD	MC	*U* value	*P* value
L3 caudal endplate	166.38 ± 7.41	159.83 ± 9.78	2.034	< 0.05
L4 cranial endplate	170.96 ± 6.25	161.92 ± 11.93	2.353	< 0.05
L4 caudal endplate	166.56 ± 8.18	158.44 ± 14.02	2.522	< 0.05
L5 cranial endplate	172.35 ± 5.57	164.63 ± 9.25	3.835	< 0.01
L5 caudal endplate	163.80 ± 7.14	156.81 ± 11.68	3.449	< 0.01
S1 cranial endplate	171.00 ± 7.48	166.57 ± 9.02	3.554	< 0.01

## Discussion

### Main findings

As far as we know, this imaging study was performed for the first time to compare the differences in lumbar sagittal parameters of LDD with/without MII. In this study, a variety of commonly used and individualized measurement parameters were used for a more comprehensive evaluation of lumbar changes on X-ray. The results of lumbar sagittal parameters revealed that LDD combined with MII had a more severe degree of lumbar degeneration than LDD alone and MII may be the severe radiographic representation in the process of lumbar degeneration. Moreover, the overconcentration of load caused by the smaller LL, SS, and IVA may be a reasonable explanation to answer why MCs are more common at the L5/S1.

### Prevalence of MCs

Due to the differences of the study population and sample, the incidence of MCs varied greatly. The result of this study indicated that the incidence of MII among patients with LDD with grades 3–5 of the Pfirrmann classification was 29.81%, which was higher than other relevant studies [17, 18]. The main reason is that most of the patients with the moderate to severe disc degeneration were recruited in our study. Of course, we could not rule out the effects of the other factors that we did not have the statistical analysis in this study, such as age, BMI, career, and lifestyle.

The distribution of MII mainly occurred at the lower two lumbar levels among the different lumbar segments [17]. Also, MCs were more likely to be observed at L5/S1 than L4/5, as reported by a recent systematic review [19]. The lower lumbar levels would be prone to bear the higher mechanical loading than the upper lumbar spine [17].

Studies have shown that the prevalence of male with MCs was higher than that of female [20]. This may be associated with a higher rate of males engaging in moderate or heavy physical work that led the lumbar spine to bear more repetitive stress loads than females. Oppositely, Xiao et al. [18] reported that although there was no statistical significance in gender, females had the higher likelihood to be with MCs than males. This study came to the same conclusion as they did. The possible reason why the rate of females with MII was higher than males could be obtained that it may be associated with osteoporosis which was caused by changes in hormone levels of the female patients at the age of high morbidity of MCs.

### Sagittal parameters

The LL, SS, and IVA formed by the lumbar curvature that is one of the key physiological arch in maintaining the posture of the human spine are the important indicators for the imaging measurement of the lumbar spine at present. There were only few studies which reported the relationships between lumbar sagittal parameters and MCs. Farshad-Amacker et al. [21] performed a long-term follow-up study to look for the predictors for the development of lumbar degeneration in terms of LDD and MCs, but they did not find the relationships between LL, SS, and the development of MCs. However, another study [15] with the contradictory results reported that the endplates with MCs in degenerative thoracolumbar/lumbar kyphosis were negatively correlated with LL (*r* = − 0.562, *P* = 0.012) and SS (*r* = − 0.46, *P* = 0.048). Obviously, the different conclusions of the above studies were based on different research subjects. Our study found that patients with MII on the basis of LDD had smaller LL and SS angles, which means that patients with MII tend to straighten the whole lumbar spine. The straightening of the lumbar vertebral curvature is the protective response so that the human body can well adapt to the degenerative changes of the lumbar spine. Almost all of the MCs were associated with the degeneration of lumbar structures, and the straightening of physiological curvature would inevitably increase the stress load on the vertebral endplate, leading to the occurrence of MCs.

In general, IVA and LL maintain a positive correlation. In our study, patients with MII had significantly smaller IVA and LL compared with patients in the DD group. However, a significant difference only occurred at the segment of L5/S1 between both groups compared with the IVA at other lumbar levels. The smaller IVA in the MC group allows the patient to concentrate more stress on the endplate while standing. Therefore, combined with the above comparison of LL and SS, this may explain the reason why the L5/S1 segment is more prone to MCs on the basis of lumbar degeneration.

The loss of intervertebral height on the lumbar radiograph is a common clinical sign of LDD, which has been reported as a positive correlation with MCs, whether by semi-quantitative or quantitative measurement [22, 23]. Their reports were consistent with our results of IHI. However, previous studies [3, 4, 24] have revealed that MCs always occurred at sites of disc degenerative disease. Therefore, the lack of a control group with disc degeneration may lead to the statistical error in those studies. In addition, because the intervertebral height is influenced by the different population, age, sex, BMI, posture, and so on, the results of the direct measurement are not superior to that of IHI, which is considered to be individualized in measuring the height of intervertebral space [25]. The lower IHI in the MC group suggests that MII plays a positive role in the process of lumbar degeneration.

The endplate is curved in the normal lumbar spine. With the aggravation in lumbar disc degeneration, the endplate tends to flatten itself to adapt the biomechanical changes [26]. This self-protective mechanism can shift the stress load from the central region of the endplate to the surrounding endplate, so as to decrease the damage of the vertebral body and endplate [18]. It also explains the reason for the increase of the average ECA in the DD group in this study. However, in the MC group, the cranial and caudal ECA of the patients were significantly smaller than the DD group, but this did not indicate that the MII had the effect of remodeling the normal morphology of the vertebral endplates. Conversely, this may be a sign of partial endplate and/or vertebral collapse caused by the inflammation and repetitive pressure loading. Above, the results could allow us to believe that the reduction in ECA may be a more severe sign of lumbar degeneration for the patients with MII.

### Limitations

As with any clinical study, our study has certain limitations. Firstly, a relatively small sample, especially in the MC group, may have decreased the statistical power. Secondly,it is regrettable that we did not set up more detailed subgroups due to the limitation of small sample. In addition, because of the limitations of patient’s economy and local medical insurance policies, the lack of whole spine radiographs has forced us to abandon the measurement and analysis of pelvic parameters. Therefore, a large sample, multicenter imaging study would be more helpful to analyze comprehensively the difference in the spinopelvic parameters between LDD alone and LDD combined with MCs and the different types of MCs.

## Conclusions

The present study indicated that the prevalence of MII in degenerative lumbar diseases in this study was 29.81%, with L5/S1 being the most common level. Compared with LDD alone, the MC group with smaller lumbar curvature (LL and SS), narrower intervertebral height (IHI), and more severe damage of endplate and vertebral body (EVA) suggested that MII has a severe radiographic representation in the process of lumbar degeneration. Also, our study reinforces the evidence for those studies that reported the positive relationship between MCs and lumbar degeneration, and the overconcentration of load caused by the small LL, SS, and IVA may be a reasonable explanation to answer why MCs are more common at the L5/S1. Therefore, we should pay much attention to the lumbar sagittal parameters in order to well understand the development of MCs in the clinical work.

## Data Availability

The datasets generated and analyzed during the current study are available from the corresponding author on reasonable request.

## Abbreviations

ECA: Endplate concave angle
ICC: Intraclass correlation coefficient
IHI: Intervertebral height index
IVA: Intervertebral angle
LDD: Lumbar disc degeneration
LL: Lumbar lordosis
MCs: Modic changes
MII: Modic type 2
MRI: Magnetic resonance imaging
SS: Sacral slope
